# Characterization and genomics identification of key genes involved in denitrification-DNRA-nitrification pathway of plant growth-promoting rhizobacteria *(Serratia marcescens* OK482790)

**DOI:** 10.1186/s12866-023-02941-7

**Published:** 2023-08-05

**Authors:** Marwa A. Hamada, Elham R. S. Soliman

**Affiliations:** Botany and Microbiology Department, Faculty of Science, Helwan University, Helwan, Egypt

**Keywords:** Ammonia production, HCN production, Nar genes, NirBD gene, 16S rDNA, Nitrate/nitrite reduction, Lytic enzymes, Siderophore production

## Abstract

**Background:**

A wide variety of microorganisms, including bacteria, live in the rhizosphere zone of plants and have an impact on plant development both favorably and adversely. The beneficial outcome is due to the presence of rhizobacteria that promote plant growth (PGPR).

**Results:**

In this study, a bacterial strain was isolated from lupin rhizosphere and identified genetically as *Serratia marcescens* (OK482790). Several biochemically and genetically characteristics were confirmed in vitro and in vivo to determine the OK482790 strain ability to be PGPR. The in vitro results revealed production of different lytic enzymes (protease, lipase, cellulase, and catalase), antimicrobial compounds (hydrogen cyanide, and siderophores), ammonia, nitrite, and nitrate and its ability to reduce nitrate to nitrite. In silico and in vitro screening proposed possible denitrification-DNRA-nitrification pathway for OK482790 strain. The genome screening indicated the presence of nitrite and nitrate genes encoding Nar membrane bound sensor proteins (NarK, NarQ and NarX). Nitrate and nitrite reductase encoding genes (*NarI, NarJ, NarH, NarG* and *NapC/NirT*) and (*NirB, NirC*, and *NirD*) are also found in addition to nitroreductases (NTR) and several oxidoreductases. In vivo results on wheat seedlings confirmed that seedlings growth was significantly improved by soil inoculation of OK482790 strain.

**Conclusions:**

This study provides evidence for participation of *S. marcescens* OK482790 in nitrogen cycling via the denitrification-DNRA-nitrification pathway and for its ability to produce several enzymes and compounds that support the beneficial role of plant-microbe interactions to sustain plant growth and development for a safer environment.

**Supplementary Information:**

The online version contains supplementary material available at 10.1186/s12866-023-02941-7.

## Background

Soil consists of diverse microscopic life forms such as actinomycetes, algae, bacteria, fungi, nematodes, and protozoans. But the rhizosphere is the most heavily inhabited part of the soil because plant roots secrete a wide variety of nutrients that draw microorganisms there. The interactions between bacteria and plants can be classified into three categories: neutral, negative or positive [1]. The majority of rhizobacteria living in and around plants are in a communalistic relationship, however, some rhizosphere bacteria, known as phytopathogens, can actually stunt a plant’s development and disrupt its physiology [2]. The positive effect of the rhizosphere bacteria may be direct and indirect on the plant growth. The direct mechanisms are represented in affecting the balance of plant’s growth, enhancing plant’s nutritional status, and stimulating systemic disease resistance mechanisms. Indirect mechanisms are related to biocontrol, including antibiotic production, chelation of available Fe in the rhizosphere, and synthesis of extracellular lytic enzymes [3]. In general, beneficial bacteria are usually referred to as plant-growth-promoting rhizobacteria or PGPR [4, 5, 3, 6]. PGPR include members of several genera, e.g., *Agrobacterium, Arthrobacter, Alcaligenes, Azotobacter, Acinetobacter, Actinoplanes. Bacillus, Frankia, Pseudomonas, Rhizobium, Micrococcus Streptomyces, Xanthomonas, Enterobacter, Cellulomonas, Serratia, Flavobacterium, Thiobacillus*, etc. [5]. Due to the different metabolic activities of these bacteria in the soil and their effect on plant growth and development, they can be grouped as biofertilizers, phyto-stimulants, and biopesticides [6, 6, 8, 1].

Genes involved in the manufacturing of phytohormones such indole-3-acetic acid (IAA), acetoin synthesis, lytic enzyme production, siderophores, nitrate reduction, and phosphate solubilization were found in *S. marcescens’* genome. The advantageous interactions it has with plants are predicated on the genetic basis underlying its metabolic properties [7]. Both root length and dry weight increased significantly after inoculation with *S. marcescens* IRBG500 in the rice variety IR72, but total N content did not [8]. *Petunia* and *Impatiens* recovered more quickly from water stress after being treated with *S. plymuthica* MBSA-MJ1, which enhanced the quantity of flowers produced by *Petutnia* and improved their visual quality. Furthermore, MBSA-MJ1 was shown to be a motile, modest antibiotic-resistant, and osmotic stress-tolerant bacteria through in vitro characterizations [9].

Bacteria of the genus *S. marcescens* have been classified as heterotrophic nitrifiers and aerobic de-nitrifiers’ [10, 11]. 

The standard classifications for nitrogen cycle conversions include assimilation, ammonification, nitrification, denitrification, anaerobic ammonium oxidation (anammox), and nitrogen fixation [12]. Well-known biological nitrogen removal processes include nitrification and denitrification, both of which are microbially driven [13]. In the nitrification process, ammonia (NH_3_) is oxidized by ammonia-oxidizing bacteria (AOB) and/or ammonia-oxidizing archaea (AOA) into nitrite (NO_2_), which is subsequently oxidized by nitrite-oxidizing bacteria (NOB) in the presence of oxygen to produce nitrate (NO_3_) [14]. By using anaerobic respiration, heterotrophic microorganisms facilitate a process called denitrification. Nitrate-reducing bacteria are crucial organisms because of their role in converting nitrate to dinitrogen gas (N_2_). Denitrification begins with the reduction of nitrate (NO_3_) to nitrite (NO_2_) via the action of nitrate reductase (NAR) and/or (NAP) enzymes located in the membrane and periplasm, respectively [15]. After that, nitrite (NO_2_) is reduced to nitric oxide (NO) by nitrite reductase (NIR), then to nitrous oxide (N_2_O) by nitric oxide reductase (NOR), and finally to nitrogen (N_2_) by nitrous oxide reductase (NOS) [16]. Denitrification and Dissimilatory Nitrate Reduction to Ammonia (DNRA) are two microbial anaerobic respiration processes that compete in the environment for nitrate and nitrite. When nitrate is broken down by denitrification, the nitrogen is released into the air as dinitrogen gas and traces of the gaseous intermediates nitric and nitrous oxide, while DNRA keeps the nitrogen in the habitat in the form of ammonium [17].

The objectives of the current study were to characterize a *S. marcescens* strain isolated from the rhizosphere of *Lupinus* as a PGPR through in vitro screening of its production to different lytic enzymes, antimicrobial agents and its role in nitrogen cycling. Genome screening for the current available databases was performed to identify and characterize the possible genes that may be involved in nitrogen cycling by *S. marcescens*. In vitro verification for the detection of these identified genes in our strain was performed. Additionally, the ability of using the current strain as a PGPR was verified in pot experiment for wheat.

## Results

### Morphological and molecular identification for the rhizosphere bacterial strain

**Fig. 1:**
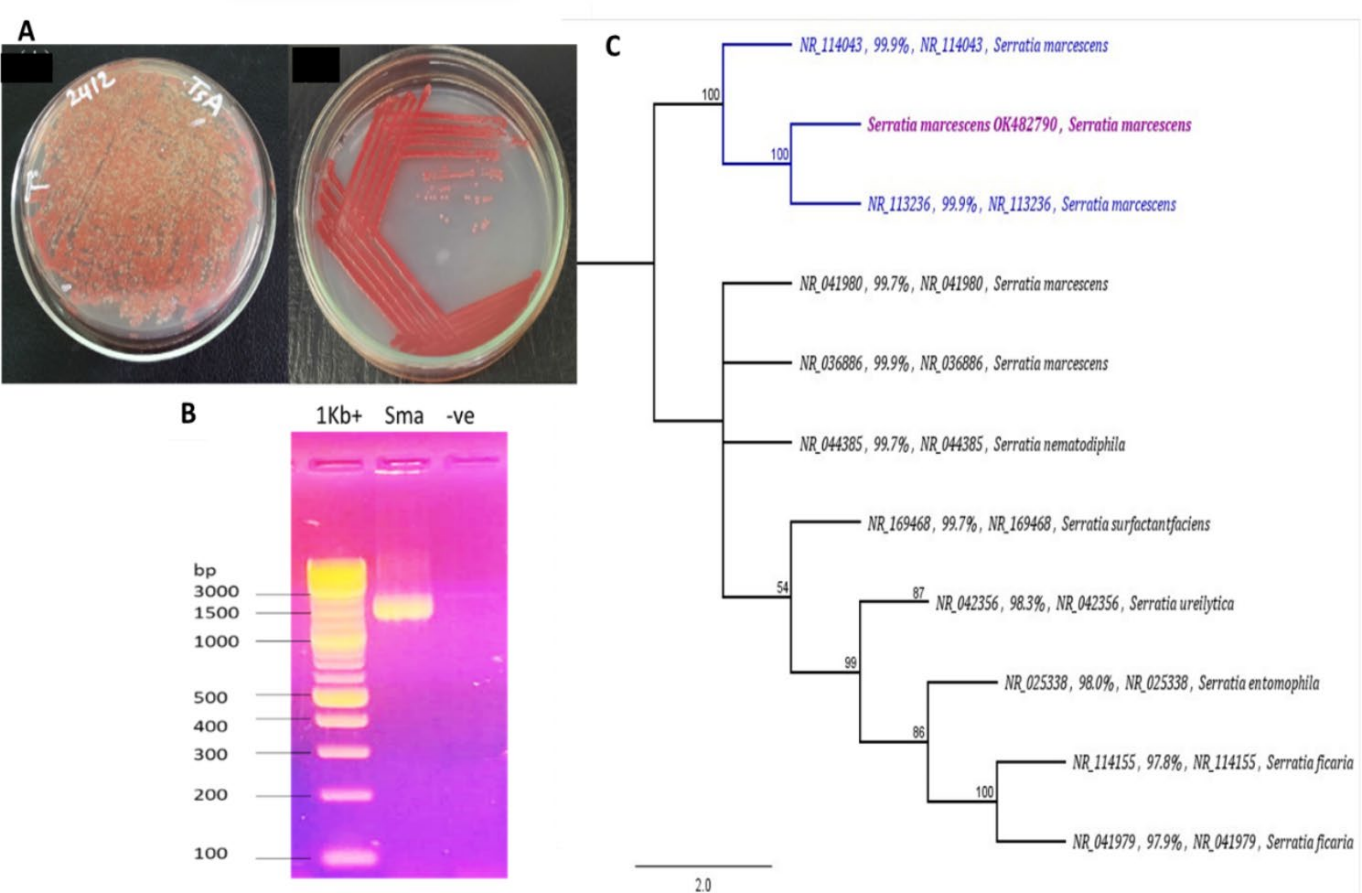
Morphological and molecular identification of *Serratia marcescens* isolated from *Lupinus* rhizosphere. **A** The growth of *S. marcescens* strain on TS agar, showing the typical morphological feature for *S. marcescens. ***B** Gel electrophoresis image for 1500 bp of 16 S rRNA encoding gene PCR fragment. Sma refers to the amplified product, -ve refers to negative control, no amplifications confirm no PCR contamination. 1 kb + refers to DNA ladder (*BioLabs*). **C** Phylogenetic tree of the 16S rRNA encoding gene amplified from the current strain. The phylogeny indicated that the sequence of the current strain; labelled in bold purple font color, belongs to *Serratia marcescens*

The bacterial strain used in the current study showed red-pigmented colonies on TSA plate. The strain was confirmed as *S. marcescens* by the 16S rDNA sequences alignment to the NCBI GenBank and the constructed phylogenetic tree. The nucleotide sequences showed 99.9% identity and query coverage homology to *S. marcescens* NR_114043 and NR_113236 strains at 100% bootstrapping value. The sequence was submitted to the NCBI GenBank and received an accession number; OK482790 (https://www.ncbi.nlm.nih.gov/nuccore/OK482790, accessed on 12 October 2021) (Fig. 1).

### In silico screening for nitrogen cycling involved genes

To determine the number of possible genes that may be involved in nitrogen cycling by *S. marcescens* OK482790, we conducted a genomic search for the full chromosome sequence of *S. marcescens* strain KS10 with accession number NZ_CP027798.1 (https://www.ncbi.nlm.nih.gov/nuccore/NZ_CP027798.1) and DB11 strain (NZ_HG326223.1) (https://www.ncbi.nlm.nih.gov/nuccore/NZ_HG326223) deposited at NCBI database, both were used as a reference strains. The search confirmed the presence of nitrite and nitrate genes’ encoding Nar membrane bound sensor proteins (*NarX, NarQ*, and *NarK*). Nitrate and nitrite reductase encoding genes are also found (*NarI, NarJ, NarH, NarG* and *NapC/NirT*) and (*NirB, NirC*, and *NirD*), respectively. Three nitroreductase enzymes were also identified that are known to be involved in the reduction of nitrogen-containing compounds. The genome coordinates of each gene with the expected product ID and function are tabulated in Table (1). It worth to note that neither nitrate nor nitrite oxidase enzymes was found in the analyzed genome, which suggests a role in denitrification rather than nitrification process.

**Table 1:** Genome coordinates, encoding protein IDs and function for genes expected to play a role in nitrogen cycling by *Serratia marcescens* according to the NCBI Gene-bank

	Gene complement	locus_tag	Protein ID	Gene product function
Nitrate/Nitrite sensor/transporter protein	273549.2274943	SMDB11_RS10660	WP_025303214.1	(**NarK**) family nitrate/nitrite MFS transporter
	2275294.2277078	SMDB11_RS10665	WP_025303215.1	(**NarX**) nitrate/nitrite two-component system sensor histidine kinase
	3048021.3049697	SMDB11_RS14285	WP_025303815.1	(**NarQ**) nitrate/nitrite two component system sensor histidine kinase
Nitrate reductase activity related genes	2251245.2251922	SMDB11_RS10580	WP_025303199.1	(**NarI**) respiratory nitrate reductase subunit gamma
	2251925.2252647	SMDB11_RS10585	WP_025303200.1	(**NarJ**) nitrate reductase molybdenum cofactor assembly chaperone
	2252644.2254188	SMDB11_RS10590”	WP_025303201.1	(**NarH**) nitrate reductase subunit beta
	2254185.2257946	SMDB11_RS10595	WP_025303202.1	(**NarG**) nitrate reductase subunit alpha
	2717928.2719088	SMDB11_RS12645	WP_025303545.1	**(NapC/NirT)** family cytochrome c
	1937451.1938215	SMDB11_RS09050	WP_004938278.1	(**Fnr**) Fumarate & nitrate reduction regulatory protein
Nitrite reductase related genes	4080213.4082762	SMDB11_RS19060	WP_025304530.1	(**NirB**) nitrite reductase large subunit
	4082759.4083085	SMDB11_RS19065	WP_025304531.1	(**NirD**) nitrite reductase small subunit
	4083102.4083902	SMDB11_RS19070	WP_025304532.1	(**NirC**) nitrite transporter
Nitroreductase gene family	1856633.1857238	SMDB11_RS08680	WP_025302894.1	NAD(P)H Nitroreductase activity
	2108509.2109060	SMDB11_RS09850	WP_004932204.1”	NAD(P)H Nitroreductase activity
	2554229.2554816)	SMDB11_RS11895	WP_038629586.1	NAD(P)H Nitroreductase activity
Putative Oxidoreductase activity	513814.3515016	SMDB11_RS16455	WP_025304151.1	(**UbiI**) gene synonym = VisC FAD-dependent 2-octaprenylphenol hydroxylase. Oxidoreductase activity

### Molecular detection of nitrogen cycling associated genes in ***S. marcescens*** OK482790

**Fig. 2:**
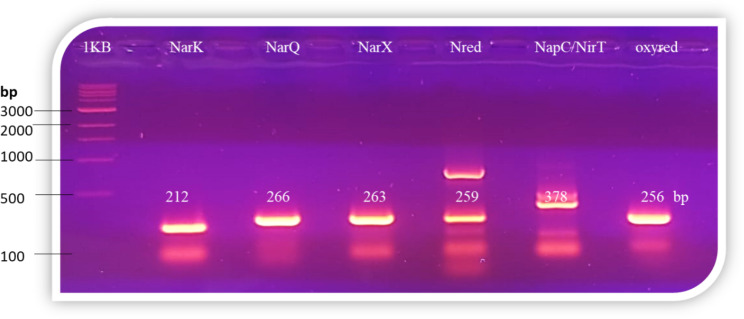
Agarose gel electrophoresis for PCR products of six nitrogen cycling related genes amplified from *Serratia marcescens* (OK482790); lanes 1 to 6 refers to the amplified products of NarK, NarQ, NarX, NTR, NapC/NirT and oxyred genes, respectively, 1KB refers to DNA ladder. The expected product sizes were indicated

Six pairs of primers were designed to confirm the presence of the in silico detected genes within the genome of our strain (OK482790). Three pairs of the primers were used to amplify three genes of Nar membrane bound sensor proteins (*NarX, NarQ*, and *NarK*), two pairs to amplify nitrate reductase and nitroreductase encoding genes (*NapC/NirT* family cytochrome c and SMDB11_RS08680, respectively) and one pair for a putative (*ubi*I). All primers were successfully amplified the expected fragment size for each detected gene (see methods section). This result confirmed the presence of the corresponding genes in our strain; *S. marcescens* (OK482790) (Fig. 2).

### In vitro PGP properties of ***S. marcescens*** (OK482790)

#### Reduction of nitrate

**Fig. 3:**
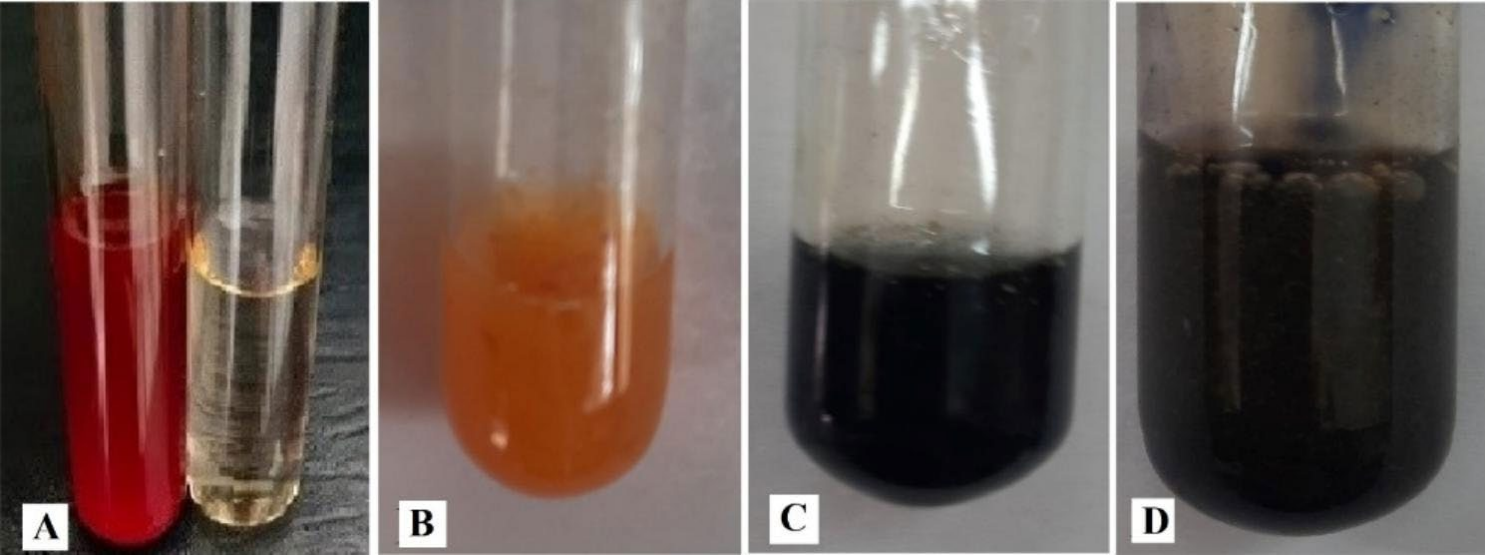
Reduction of nitrate and production of ammonia, nitrite, and nitrate by *Serratia marcescens* OK482790 strain. Where **A-** Deep red colour with sulfanilic acid and alpha-naphthylamine mixture (1:1) indicated nitrite production. **B-** Golden yellow colour with Nessler’s reagent indicated ammonia production. **C-** Deep blue colour with Trommsdorf^,^s reagent indicated nitrite production. **D-** Deep blue colour with Diphenyl amine reagent indicated nitrate production

Reduction of nitrate to nitrite was confirmed by OK482790 strain which detected by the formation of a deep red colour for nitrate medium containing culture post the addition of sulfanilic acid and alpha-naphthylamine mixture (1:1) reagent (Fig. 3A).

#### Production of ammonia, nitrite, and nitrate

Production of ammonia was determined on the peptone-water media and OK482790 strain showed positive golden yellow colour with Nessler^’^s reagent indicating that ammonia was produced (Fig. 3B). For nitrite and nitrate production, deep blue colour was produced with Trommsdorf^,^s and Diphenyl amine reagents, respectively (Fig. 3C and D).

### Production of IAA, HCN and siderophores

**Fig. 4:**
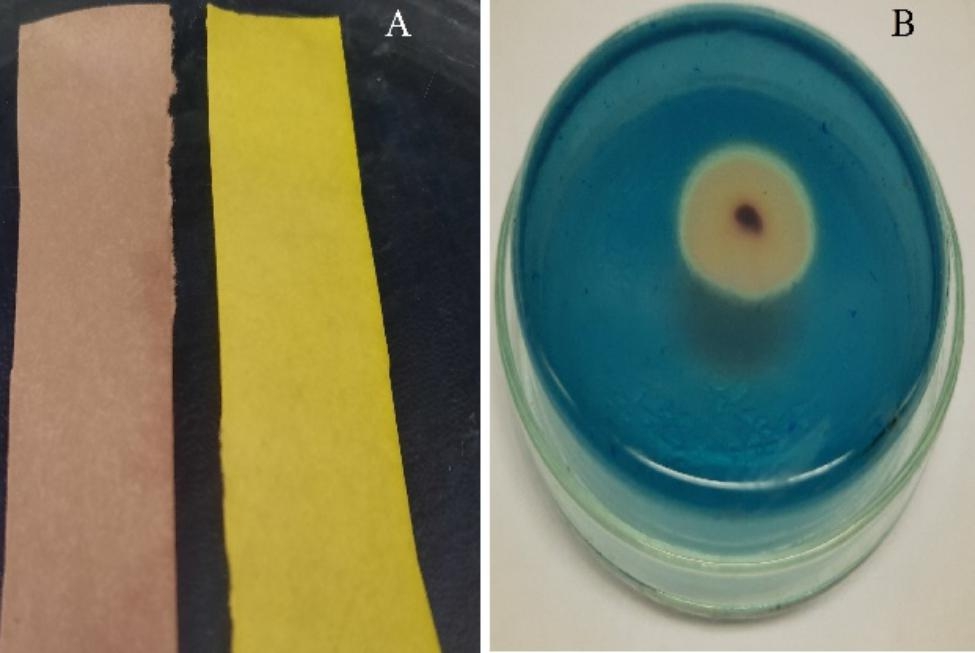
Production of hydrogen cyanide in **A** and siderophores in **B** by *Serratia marcescens* OK482790 strain

OK482790 strain was screened to produce IAA, HCN and siderophores on characteristic media as mentioned in methods section. The results appeared positive with HCN and siderophores production as indicated in Fig. (4). But appeared negative with IAA production.

### Production of cellulase, protease, lipase, catalase, and amylase enzymes

**Fig. 5:**
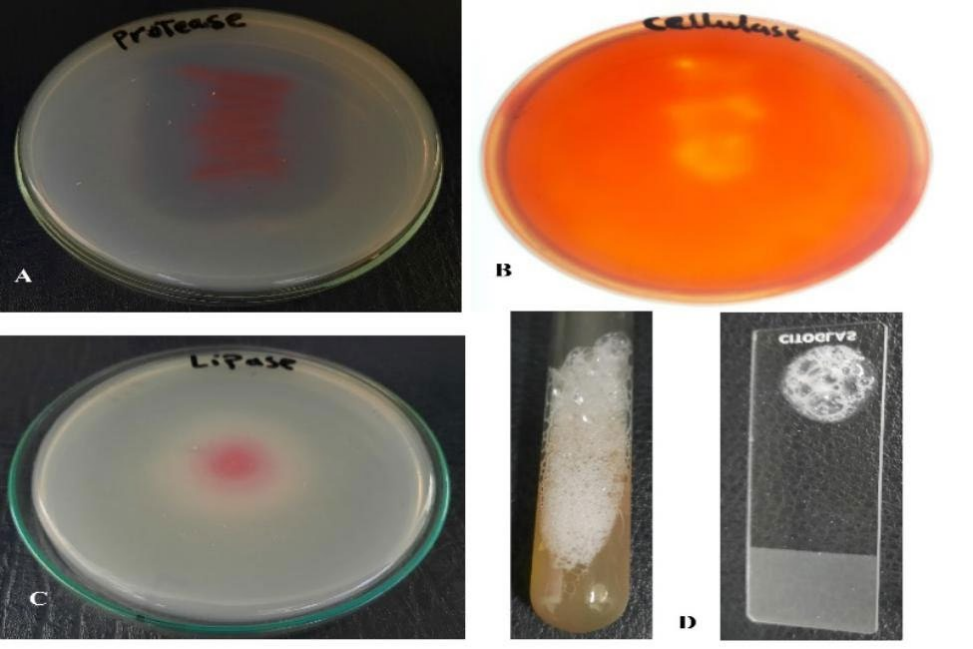
Different enzymatic activities of *Serratia marcescens* OK482790 strain. **A**) Protease **B**) Cellulase **C**) Lipase **D**) Catalase

*S. marcescens* strain OK482790 was screened for its ability to produce some lytic enzymes by assessing its activity toward some substrates such as carboxymethyl cellulose (CMC), gelatin, tween 20, hydrogen peroxide and starch. The results showed that OK482790 strain was positive with cellulase, protease, lipase, catalase. But it was negative with amylase test. The results were detected after only 24-hour for lipase, protease, catalase as indicated in Fig. (5).

### Effect of ***S. marcescens*** (OK482790) on wheat vegetative parameters

**Fig. 6:**
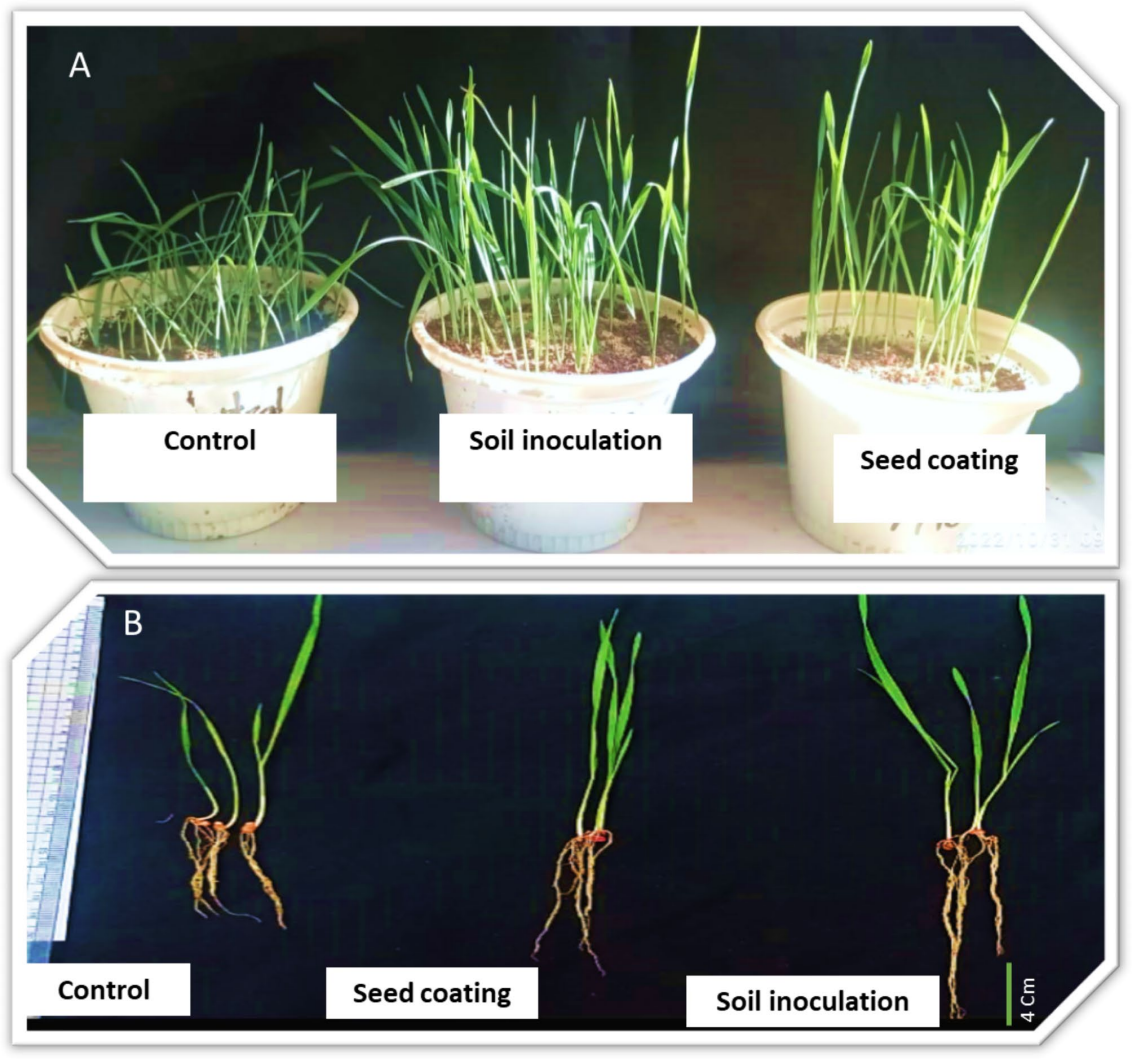
Effect of PGPR; *Serratia marcescens* (OK482790) by soil inoculation or seed coating on wheat’s shoots and roots’ heights, fresh and dry weights in comparison to uninoculated plants (the control) of two-week-old seedlings. **A-** Pot experiment of two weeks old seedlings showing the growth promoting effect of *S. marcescens* inoculation. **B-** Effect of *S. marcescens* on the length of wheat shoot and root stock

To confirm the effectiveness of using the current strain as a PGPR, a pot experiment using wheat plants was performed. *S. marcescens* (OK482790) was inoculated to soil by mixing a fresh bacterial suspension otherwise was inoculated by seed coating with the bacterial suspension. Both inoculation methods were effective for promoting wheat growth (Fig. 6). Shoot and root lengths were greatly improved by bacterial inoculation and was more effective in case of soil inoculation reached up to 13.3a ± 0.46 and 11.06a ± 0.77, respectively, with 30.5 and 109.07% increase over the control plants (uninoculated) (Fig. 6b). As well as soil inoculation showed the most promoting effect on shoot and root fresh and dry weights. A significant increase reached up to 295 and 172% in comparison to control fresh and dry shoots (Table 2).

**Table 2:** Effect of *S. marcescens* (OK482790) inoculation, to soil or by seed coating on wheat’ shoots and roots height, fresh and dry weights, in comparison to uninoculated plants (Control). Data represents the means of 20 independent replica ± SD

	Shoot length	Root length	Shoot fresh wt	Root fresh wt	Shoot dry wt	Root dry wt
Control	10.19^b^ ± 0.24	5.29^c^ ± 0.27	0.040^b^ ± 0.0067	0.088^a^ ± 0.039	0.0068^c^ ± 0.0007	0.022^a^ ± 0.018
Seed coating	12.72^a^ ± 0.35	7.32^b^ ± 0.31	0.093^ab^ ± 0.0047	0.088^a^ ± 0.0047	0.0156^b^ ± 0.0007	0.024^a^ ± 0.0034
Soil inoculation	13.3^a^ ± 0.46	11.06^a^ ± 0.77	0.158^a^ ± 0.041	0.10^b^ ± 0.0046	0.0185^a^ ± 0.0009	0.024^a^ ± 0.0011
LSD at 0.05%	2.53	2.03	0.118	0.089	0.002	NS
NS: indicates no significance						

## Discussion

The rhizosphere bacteria play a critical role in the acquisition of various functional features required for plant fitness and increase the plant’s functional repertoire [3, 18]. Here in a lupin rhizosphere-isolated bacterial strain was identified by 16S rDNA sequencing as *Serratia marcescens* (OK482790) was subjected to genome data analysis and different biochemical screening to evaluate its potential use as a plant growth promoting rhizobacteria (PGPR).

One of the PGP characteristics of the OK482790 strain was ammonia generation. Nitrogen (N) and its derivative ammonia promote plant growth because they directly feed N to plants [19]. Ammonia can be produced through several processes, including nitrite ammonification, the degradation of various amino acids found in food or complex media, the decarboxylation of amino acids to produce biogenic amines in addition to ammonia, deamination, and the urease-mediated hydrolytic degradation to ammonia. Consequently, several enzymes, including lyases, amino acid and nucleotide deaminases, nitrilases, nitrite reductases, pyridoxamine phosphate oxidases, and amino acid deaminases, may be implicated in the production of ammonia [20]. Nitrate, nitrite, ammonia, and nitrogen are main sources of nitrogen and obtained within two processes, nitrification, and denitrification as a part of nitrogen cycle. Reduction of nitrate to nitrite by nitrate reductase and from nitrite to ammonia (DNRA-pathway) was evaluated in *S. marcescens* (OK482790) in silico and in vitro [23, 24]. The naturally occurring microbial communities in a plant’s rhizosphere provide a cost-effective and ecologically friendly solution to soil nitrate contamination in favor of nutrient-rich soil for improved plant growth. Nitrogen is one of the most important elements for plant growth, and plants absorb it from the soil as nitrate (NO_3_), nitrite (NO_2_), and ammonium (NH4^+^). Where nitrification occurs and is taken by plants, nitrate is the major form of available nitrogen under aerobic conditions [11, 21]. The isolated *S. marcescens* (OK482790) was able to produce ammonia and reduce NO_3_ aerobically. To understand the possible pathway involved in the production of these compounds, an *in-silico* genome analysis was performed to identify the possible enzymes encoding genes that could be associated.

It is worth noting that neither nitrate nor nitrite oxidase enzymes existed in the analyzed genome, suggesting a role in denitrification rather than nitrification. The nitrification process is initiated by ammonia oxidation to nitrite then nitrate by the action of ammonia monooxygenase (*amoA)* and nitrite oxidase respectively. PCR amplification using gene specific primers targeting alpha subunit of ammonia monooxygenase gene (*amoA*) and nitrite oxidase (*NxrA*) failed to produce any reproducible band which confirmed the in silico data. PCR amplification of *amoA*, and *nxrA* genes provided evidence of nitrification ability by a thermotolerant *Brevibacillus Agri* N2 strain isolated from sewage sludge [21].

The in silico genome search revealed the presence of nitrite and nitrate genes encoding Nar membrane bound sensor proteins (*NarX, NarQ*). Nitrate and nitrite reductase encoding genes were also found (*NarI, NarJ, NarH, NarG* and *NapC/NirT*) and (*NirB, NirC*, and *NirD*), respectively. It was proposed that nitrate reductase was responsible for the first step of denitrification of NO_3_^−^ to NO_2_^−^, and two types of it might have existed: one was membrane-bound nitrate reductase (Nar) and the other was periplasmic nitrate reductase (Nap). Denitrifying bacteria respond to nitrate/nitrite through sensor proteins: NarX, NarQ which may be under the control of the Fumarate Nitrate reductase Regulator protein: FNR that bind DNA to control induction of the *Nar* and *Nap* operons [22–24]. *S. marcescens* is a proteobacteria in the γ subdivisions, which commonly confined the presence of *NarX* and *NarQ* genes within their genomes like *Escherichia*, *Salmonella*, *Klebsiella*, and *Pseudomonas* species. For nitrogen dissimilation, nitrate must be transported into the bacterial cells by nitrate/nitrite transporter protein: NarK. The *E. coli* chlC locus includes the *NarK* gene that encodes a nitrite efflux porter [25, 26] while NarK protein in the denitrifying bacterium *Pseudomonas aeruginosa* PAO1 displayed a role in nitrate import and nitrite export to the periplasm [27]. The imported NO_3_^−^ to the periplasm would be reduced by the action of membrane bound nitrate reductase (Nar) enzymes. Nar enzyme is composed of three subunits: a catalytic a subunit (NarG), a soluble b subunit (NarH), and a membrane biheme b quinol-oxidizing g subunit (NarI). A delta polypeptide (NarJ) is co-existed, but it is not a part of the apoenzyme, but it activates it prior to its membrane attachment [23, 28]. The NapC/NirT family cytochrome c is a periplasmic nitrate reductase which was identified in *S. marcescens’ in silico* genome analysis. Its activity generates nitrite that could be used as a nitrogen source or as a substrate for anaerobic respiration depending on the organism. Mutational analysis in *Rhodobacter sphaeroides* proves a role of NapC in the electron transfer to the periplasmic enzyme complex [29, 30]. While, denitrifying bacteria electron transport chain utilizes most of the aerobic electron transport chain, diverting electrons from cytochrome *c* to nitrate [31]. Nap genes of the *E. coli* are induced anaerobically by fumarate nitrate reduction regulatory protein (Fnr), that was also identified in *S. marcescens* genome. This is suggesting a similar regulatory pathway for NapC as well as Nar in *S. marcescens* by Fnr [22].

**Fig. 7:**
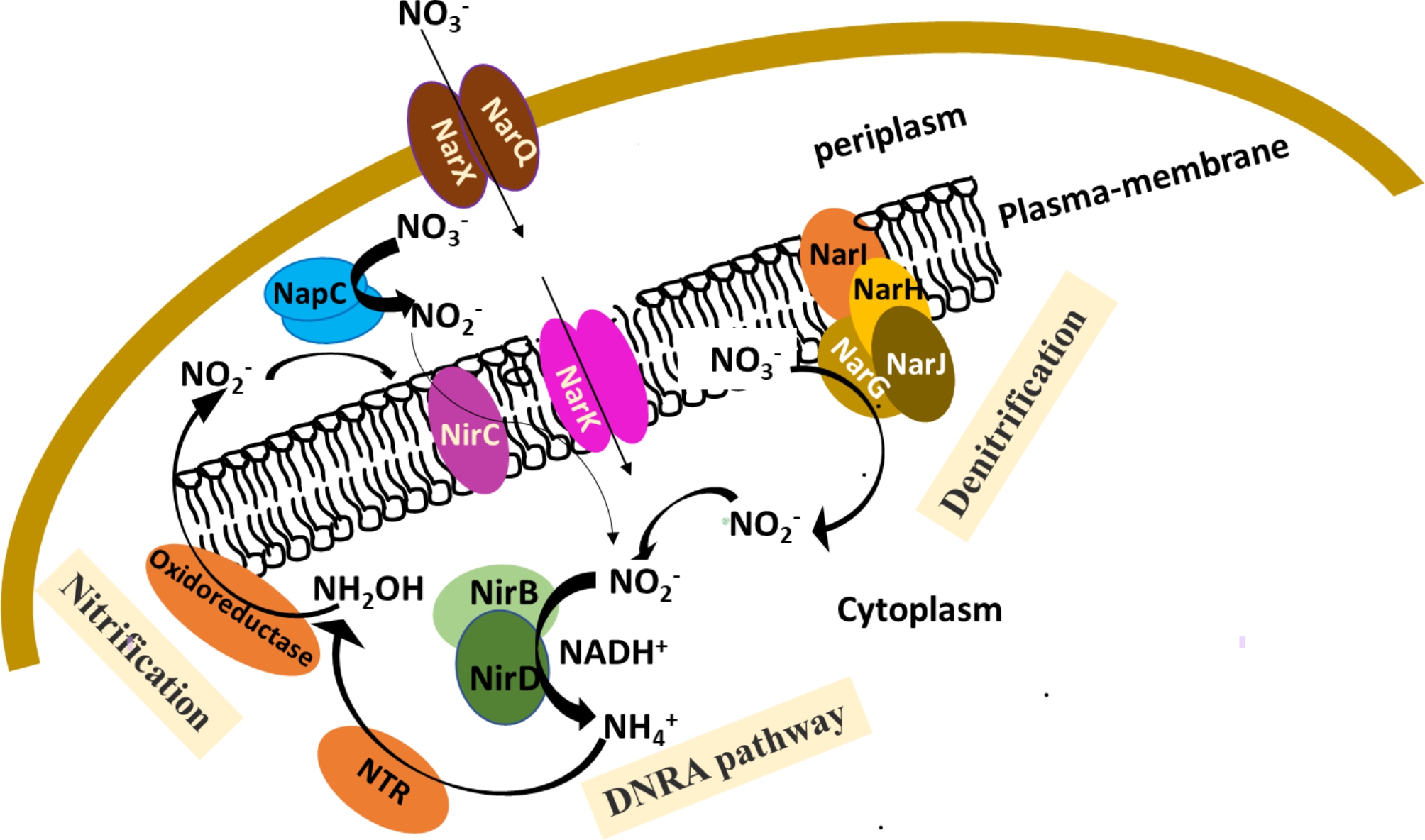
Possible denitrification-DNRA-nitrification pathway of *Serratia marcescens* (OK482790) strain based on genome and biochemical screening analysis

Nitrite reduction is the second step in denitrification process which is carried out by nitrite reductase (Nir). In *S. marcescens* genome, there are three Nirs, two of them are structural unit of the respiratory nitrite reductase enzyme; NirB and D and one nitrite transporter (NirC). Complementation experiments established that the NirB, NirD and CysG polypeptides are essential and sufficient for NADH-dependent nitrite reductase activity in *E. coli*. P-cysG polypeptides are located within the *NirC* gene [32]. NO_2_ could be transported to the cytoplasm by Nitrite transporter encoding protein NirC. In betaproteobacteria, a cytoplasmic NADH-dependent enzyme encoded by the *NirB* gene catalyzes the reduction of nitrite to ammonium to detoxify the nitrite that accumulates in anaerobic nitrate-respiring cells and to regenerate NAD. Ammonium generated by this enzyme can be assimilated, the process is termed nitrite dissimilation [33]. In *Streptomyces coelicolor* A3(2), NirBD was important to retain the nitrogen oxide cycle (NO_3_^−^→NO_2_^−^→NO→N_2_O^−^) and nitrite removal system by regulating NO homeostasis and to complete NO signaling [34]. In another hand, the copper nitrite reductase (NirK) or cytochrome cd1 nitrite reductase (NirS) are detected in many denitrifier communities however, NirK /NirS are catalyzing the reduction of nitrite to nitric oxide (NO), then to nitrous oxide (N_2_O) that finally reduced to N_2_ [35]. As N_2_O is toxic to the bacterial cell so the nitrite reduction by NirS/NirK occurs in the periplasm. These two genes are considered markers for denitrifying bacteria although they rarely coexist in the same bacterium species. Therefore, they recently used as a marker to distinguish ecologically distinct denitrifying groups [35]. In contrary NirBD encoding gene is in the cytoplasm catalyzed the reduction of nitrite to ammonia [36]. The nirBD of *Pseudomonas putida* strain Y-9 had an important role in strain growth and ammonium production. A ^15^N isotope experiment demonstrated that strain Y-9 can conduct dissimilatory nitrate reduction to ammonium (DNRA) and NirBD controls this process [37]. Wang and his coauthor (2016) have showed that *S. marcescens* strain W5 was able to transform ammonia to hydroxylamine through nitrification in the meantime was able to perform denitrification [10]. The current in silico genome analysis has revealed three possible targets for nitroreductase (NTR) which may be involved in conversation of NH_4_ to NH_2_OH. Recently, genome survey in addition to mutation analysis of NirBD in *Pseudomonas putida* Y9 proved its ability to perform nitrate assimilation, dissimilation of nitrate to ammonium by reduction and denitrification in presence of O_2_ [37]. Depending on the genome composition regarding genes involved in N_2_ cycle, it is most likely that similar pathway may exist in *S. marcescens* (Fig. 7). In addition, In vitro analysis for *S. marcescens* (OK482790) confirmed its ability to reduce NO_3_ and produce NH_4_^+^ which further validated the proposed possible denitrification-DNRA-nitrification pathway. After that, the ammonia that had been made was lost when it turned into nitrite and then nitrate, and the denitrification-DNRA pathway started all over again. The possible enzymes involved in the conversion of ammonia to N_2_ in *S. marcescens* are still to be elucidated.

The in silico genome analysis of *S. marcescens* was verified in the current strain (OK482790). In which, the gene specific primers designed based on the NCBI deposited genome sequences were successfully amplified the corresponding six nitrogen related genes (see methods section). The primers designed to amplify 259 bp of the nitro-reductase gene, had amplified an additional band, which could be a result of the presence of three nitro-reductases that share some similarity. Genome-wide identification of key enzyme-encoding genes involved in nitrogen cycle coupled with PCR validation in vitro paves the way for reconstructing entire pathway for nitrogen cycle in various organisms. Moreover, provides a theoretical foundation for discovering new genes’ encoding enzymes which could be employed as functional gene markers [38–41].

Here we showed that the isolated strain possesses some characteristics allowing it to be an effective biocontrol agent in soil. In addition to its possible ability to denitrificate-DNRA-nitrificate the soil, it could produce several lytic enzymes such as cellulase, protease, lipase and catalase. The production of all these enzymes supported its PGP ability as these enzymes could inhibit the growth of pathogenic microorganisms. Protease and cellulase are two enzymes that are thought to be crucial in the biological and physicochemical changes of soils, which are thought to promote plant growth. Through the breakdown of fungal membranes, both enzymes contribute to the biocontrol of infections [4]. Catalase is an antioxidant enzyme that was observed positive in our results. Presence of it may allow the plants to tolerate detoxification and decrease the level of some environmental stress [42]. Gupta and his coauthors reported that different lytic enzymes produced by PGPR, such as chitinases, dehydrogenases, glucanases, lipases, phosphatases, and proteases, demonstrate hyperparasitic activity, attacking pathogens by excreting cell wall hydrolases. The action of these enzymes significantly contributes to the suppression of numerous pathogenic fungi, such as *Botrytis cinerea*, *Sclerotium rolfsii*, *Fusarium oxysporum*, *Phytophthora* sp., *Rhizoctonia solani*, *and Pythium ultimum* [43]. The PGP effect of the current strain was confirmed on wheat seedlings. in which the bacterial inoculation to soil or by seed coating significantly enhanced the measured growth parameter such as, shoot, root length, fresh and dry weights as indicated in Fig. 6; Table 2. The growth promoting effect could be attributed to the improved water and nutrients uptake required by plants through the colonized rhizospheric bacteria. Additionally, this promoting effect could result from the antimicrobial activity that inhibited the pathogenic agents which can damage plant roots. Hydrogen cyanide (HCN), siderophores and prodigiosin pigment (unshown data) were examples of antimicrobial agents that were detected in *S. marcescens* (OK482790) strain and promote its biocontrol activity. Siderophores are organic molecules of low molecular weight that are created by bacteria and fungi to increase the intake of iron. They are also thought to be an effective supply of iron for plants, which would encourage plant growth [44]. Since fungal siderophores have a lesser affinity for iron that is present in soil, bacteria play a significant role in the prevention of plant diseases by iron sequestration [45]. Lurthy and his coauthors, reported that the siderophores’ Fe affinity regulates Fe competition in the rhizosphere, with ligands produced by the biocontrol agent having higher formation constants than those of the pathogen [46]. Siderophore synthesis, affects plant growth by attaching to the readily available iron form Fe^3+^ in the rhizosphere [47]. Hydrogen cyanide (HCN) is a volatile metabolite with an antagonistic vocation activity. Both HCN and ammonia could inhibit pathogens growth and fungal activity by acting as metabolic inhibitors, in addition to their roles in counteracting the negative effects induced by phytopathogenic microorganisms [4]. Agossou et al., (2016) reported that tomato bacterial canker was controlled biologically using PGPR *Pseudomonas* strains that produce HCN [48]. It is important to note that the detected promoting impact was greater when the soil was inoculated with PGPR, which was advantageous because this genus is recognized as rhizospheric bacteria that prefer to colonize around the root system, and their presence in the soil would guarantee this. Alternatively, seed coating with the bacteria could facilitate its passage through the micropyle and into the plant tissue, which may not be the optimal environment for the bacteria to carry out their function. Nevertheless, some bacteria may stick to the seed surface and colonize the soil after planting. However, their numbers would be limited relative to the desired soil colonization. Application of *S. plymuthica* increased shoot biomass and flower number of greenhouse ornamentals; *Petunia* and *Impatiens* in response to drought stress by modulating phytohormone levels, synthesize of osmo-protectants compounds, vitamins, and increase the availability of nutrients to plants [9]. Inoculation of *S. marcescens* strain JW-CZ2 significantly increased shoot height and stem diameter of tea seedlings via solubilization of inorganic phosphate, inhabiting the growth of pathogenic fungi, and production of IAA and siderophores [49].

## Conclusion

Here in a bacterial strain was isolated from lupin rhizosphere and identified morphologically and genetically as *S. marcescens* (OK482790). In silico genomic analysis for key genes involved in a putative nitrogen cycling of *S. marcescens* were identified and detected in vitro. A potential denitrification-DNRA-nitrification pathway was hypothesized based on both genome and biochemical screening (Fig. 7).

The OK482790 strain could produce many lytic enzymes, such as cellulase, lipase, and protease, and produces ammonia, HCN, siderophores, and reduces nitrate and nitrite, suggesting a function in plant growth promotion. Soil inoculation of (OK482790) strain had a substantial growth-promoting impact on wheat plant, as evidenced by an increase in plant fresh and dry biomass and seedling length. These results indicated that the OK482790 strain has the potential to be used as a PGPR.

### Methods

#### Isolation and characterization of rhizosphere bacteria

Bacterial isolate used in the current study was collected from the rhizosphere of lupin (*Lupinus* L.) that was collected from the plant nursery of Helwan university (29°52’10.1"N 31°19’12.2"E) with permission. The bacterial isolation was carried out according to [50] as follows: Ten grams from the rhizosphere of lupin were suspended in 90 ml sterile H_2_O contained in 250 ml Erlenmeyer flask and shaken vigorously for 2–3 min. The soil suspensions were then serially diluted (10^− 2^:10^− 6^) with a sterile H_2_O. 100 µl from each tube was spread on tryptic soy agar (TSA) plates. Plates were then incubated at 28˚C for 24 to 48 h. The isolate was picked up and sub-cultured on tryptic soy agar and purified. Then, was maintained on TSA slants and stored at 4^o^C and sub-cultured monthly.

### PCR reaction and sequencing

The total genomic DNA was isolated from overnight grown pure bacterial culture according Vejlupkova, and Fowler protocol [51]. The isolated DNA was used as a template for PCR reaction using the corresponding primer for each detected gene (Table 3). The PCR reactions were carried out in 50 µl reaction volume using Cosmo DNA polymerase red master mix (Willowfort:WF-1,020,201) according to manufacture instructions in Biometra thermal cycler (Germany). The cycling condition for each primer pair was as follow: An initial denaturation cycle at 95°C for 3 min was performed followed by 35 constitutive cycles of denaturation at 95°C for 20 s, annealing (corresponding Tm/gene) for 20 s, extension at 72°C for 20 s. Finally, an extended extension cycle was performed at 72°C for 10 min. The amplified amplicons were detected in 1.2% agarose gel followed by gel purification using PCR-M clean up system (VIOGENE cat# PF1001) according to manufacturer’s protocol. The purified PCR amplicons were sequenced using the forward primer for strand sequence initiation at GATC Company applying Sanger technology. Isolate identification was verified through NCBI nucleotide Basic Local Alignment Search Tool (https://blast.ncbi.nlm.nih.gov/Blast.cgi). The phylogenetic tree is constructed using the UPGMA tree build method with 100 bootstrapping in Geneious 11.1.5 software [52].

***In silico*** **screening for nitrogen cycling involved genes**

Genome composition of reference strains *Serratia marcescens* KS10 and DB11 *(*NZ_CP027798.1 and NZ_HG326223.1) were in silico screened to identify the key enzymes that might be involved in nitrogen cycling in soil. The genes encoding nitrate/nitrite sensors, transporters, and reduction in *Serratia marcescens*, their chromosomal position and their protein product IDs and function were retrieved from NCBI database (https://www.ncbi.nlm.nih.gov/nuccore/NZ_HG326223).

### Molecular identification and detection for nitrogen cycling involved genes

Both 27 F and 1492R primers were used to amplify ~1500 bp fragment of the 16S rRNA encoding gene that was used for molecular barcoding the current isolate [53]. Six pairs of primers were designed using NCBI primer design tool (Primer designing tool; nih.gov) to detect the presence of nitrogen cycling involved genes within the genome of our isolate. Each target gene sequence was retrieved from the NCBI database and used as a template to design the corresponding primers. The primers sequences, Tms and the expected amplicon size for each corresponding gene are listed in Table (3).

**Table 3:** List of the designed primers and their Tms, IDs, sequences, the amplicon ID and length

Primers ID	Tm ^o^C		primer sequence 5’ > 3’		Amplicon ID according to database	Amplicon length/ bp
27 F		50	AGAGTTTGATCCTGGCTCAG		16 S rDNA gene	1500
1492R			GGTTACCTTGTTACGACTT			
NarX-F		55	GGATGAAATGGCGTCGCTTG		nitrate/nitrite two-component system sensor histidine kinase, *NarX*	268
NarX-R			TTCCCGGCTGTCGTTTTCAT			
NarQ-F			CTGTTCGGCATCGTGATACT		nitrate/nitrite two-component system sensor histidine kinase, *NarQ*	266
NarQ-R			TGCAGCGCCAGATAGTGGTT			
NarK-F			CCGTAGAAGGCGATGAACGA		NarK family nitrate/nitrite MFS transporter	212
NarK-R			ACGCAATTTCCGGAGGTCGT			
oxyred-F			CAGATCGTTCAGGCCCATCG		Putative Oxidoreductase, *UbiI* “SMDB11_RS16455”	256
oxyred-R			CGAGCTGATTTCCGAACTGC			
NTR-F			ATGAATTGCTGCACCGGCTC		Putative nitroreductase “SMDB11_RS08680”	259
NTR-R			ACGTGATGACGCTCGAAGAG			
NapC/NirT-F			AACCTGCATCGATTGCCACA		NapC/NirT family cytochrome c (nitrate reductase)	378
NapC/NirT-R			GTTTCCGCATCGGTCTGAGTC			
AmoA-1 F			GGGGTTTCTACTGGTGGT		alpha subunit of ammonia monooxygenase gene	-
AmoA-1R			CCCCTCKGSAAAGCCTTCTTC [K = G or T; S = G or C]			
NxrA- F			CAGACCGACGTGTGCGAAAG		nitrite oxidase, *NxrA*	-
NxrA- R			TCCACAAGGAACGGAAGGTC			

### Evaluation of PGPR characteristics in vitro

#### Nitrate reduction assay

According to [54] with a slight modification, the isolated strain was tested for nitrate reduction as the following method: A loopful of previously cultured strain (24 h- age) was inoculated in the prepared broth nitrate medium (beef extract 5 g, peptone 5 g, potassium nitrate 1 g, and 1000 ml distilled water). All inoculated tubes were incubated at 28ºC for 3–5 days and the results were detected as deep red colour by adding sulfanilic acid and alpha-naphthylamine mixture (1:1).

#### Ammonia production assay

The isolated strain was tested to produce ammonia according to [55]. Fresh culture (24-h age) was inoculated into 10 ml peptone water medium (Biolife, Sarasota, Florida) and incubated at 28°C for 7 days. Nessler^’^s reagent (0.5 ml) was added to each tube. Development of golden yellow color was a positive test for ammonia production [56]. To prepare one liter of Nessler’s reagent (K_2_HgI_4_): dissolve 100 g of mercuric iodide and 70 g of potassium iodide in a small amount of distilled water. Add this mixture slowly with stirring to a cooled solution of 160 g of NaOH in 500 ml of distilled water. Then, dilute the mixture with water to 1000 ml [57].

#### Nitrite and nitrate production assay

According to [58], the isolated strain was tested for nitrite and nitrate production using the following method: Fresh culture (24-h old) was inoculated into a 250 ml Erlenmeyer flask containing 100 ml Stephenson’s medium (for ammonia oxidation, it contained ammonium sulphate, and for nitrite oxidation, it contained sodium nitrite) and incubated for 14 days at 28°C. The detection of nitrite and nitrate production was observed as a deep blue colour by using Trommsdorf’s and Diphenylamine reagents, respectively.

#### Hydrogen cyanide (HCN) production assay

To determine the production of HCN, A loopful of overnight culture was streaked onto nutrient agar plates supplemented with glycine (4.4 g/l). Then, the Petri dish was inverted and a sterile piece of a Whatman filter paper no. 1 impregnated with picric acid solution (yellow) (0.5% picric acid in  2% sodium carbonate) was placed on the lid. The Petri dish was sealed with parafilm and incubated at 28ºC for 96 h. The positive result of HCN production was indicated by the development of orange to red color on the paper after the incubation period [59].

#### Indole acetic acid (IAA) production assay

Screening of IAA production was carried out according to [59] as follows: 24-h age culture was streaked onto LB agar and the plates were overlaid with Whatman no. 1 filter paper. After incubation for 3 days at 28ºC, the paper was removed and treated with Salkowski^,^s reagent (1.2% FeCl_3_ in 37% H_2_SO_4_). IAA production was identified by the formation of a red halo on the paper immediately surrounding the bacterial colony.

#### Siderophores production assay

Production of siderophore was screened on chrome azurol S (CAS) agar medium according to [63] method. The fresh bacterial strain was spotted in the center of CAS agar plates. Then, the plates incubated at 28°C for 5 days. Orange color around the bacterial isolate indicates positive siderophores production.

#### Catalase test

*S. marcescens* strain OK482790 was subjected to catalase test according to [60] by slide drop method and tube method.

#### Lipase test

Production of lipase enzyme was screened on Tween-20 agar medium according to [61]. A loopful of 24-h old culture was spotted in the center of the characteristic medium. Then, the plates incubated at 28ºC overnight. The lipolytic activity was observed as a visible precipitate around the bacterial growth due to the calcium salt formed by lipase reaction.

#### Protease test

Production of protease enzyme was qualitatively screened on gelatin agar medium according to [62]. A loopful of 24-h culture was streaked in the center of the used medium. Then, the plates incubated at 28ºC for 2 days. The proteolytic activity was observed as a clear zone around the bacterial growth. To see better results, the gelatin agar plates were flooded with HgCl_2_ solution (HgCl_2_ 5.0 gm, concentrated HCl 20 ml and distilled water 100 ml). The unhydrolyzed gelatin react with HgCl_2_ forming white opacity making the clear zone clearly visible.

#### Cellulase test

Production of cellulase enzyme was determined on Bushnell Haas medium (BHM) agar plates supplemented with carboxymethylcellulose (CMC) according to [63]. A loopful of 24-h culture was streaked in the center of the BHM-CMC medium. Then, the plates incubated at 28ºC for 4 days. The detection of cellulase enzyme was observed using plate staining method according to [64]: The plates were flooded with 0.3% Congo red for 20 min. Then, the stain was poured off, and the plates were washed with 1 M NaCl. Appearance of clear zone around the bacterial growth indicated the positive cellulase result.

#### Amylase test

Nutrient agar containing 0.2% soluble starch was poured in Petri-dishes, plates were inoculated with 24 h-aged culture, incubated for 4–7 days at 28ºC and starch hydrolysis was detected by flooding plates with iodine solution [65].

### Pot experiment

In vivo pot experiment was done on wheat seeds; *Triticum aestivum* according to [66] with a slight modification, seeds were obtained from agricultural research center, Giza, Egypt and planted after permission. Plastic pots, each of 20 cm diameter and containing soil (2 peatmoss: 1 sand v/v) were used for seedlings growth. Peatmoss was purchased packaged and sterilized from the local market, and the sand was sterilized in the autoclave at 121°C and 1.5 atm for 20 min prior to use.

Three sets of seeds were grouped, the first one acted as a control group were sowed directly in the plastic pots and referred to control treatment, the second group of seeds were soaked for 1 h in a fresh suspension of *Serratia marcescens* strain OK482790 containing 2 × 10^9^ CFU/ml, then, seeds were air dried for 1 h and sowed in the plastic pots (seeds coating treatment). The third group of seeds, (soil inoculated treatment) prior to being sown in plastic containers, had their soil inoculated with a fresh suspension of *Serratia marcescens* strain OK482790 containing 2 × 10^9^ CFU/ml (10 ml of bacterial suspension/1 kg of soil). Each treatment was done in triplicate and each replica contains ≥ 15 seeds. The seedlings were grown in the open air at room temperature and all the pots were watered as required to keep the soil moist. After two weeks, seedlings of the three treatments were examined and the results were detected.

### Measurement of aerial and root height and Biomass

The height (cm) growth of the plants’ shoots and roots were measured for two weeks old seedling using a graduated ruler. The plants were harvested, and the fresh weight of the aerial and root biomass produced for each treatment was weighted. The aerial and root parts were dried in the oven at 65°C for 2 days until constant weight, then their dry weights were determined using an electronic precision balance.

### Statistical analysis

The data were subjected to one way analysis of variance (ANOVA) using SPSS package. The averages of the replica measured at the 5% probability threshold (p ≤ 0.05) were compared using LSD and Duncan’s multiple comparison test.

### Electronic supplementary material

Below is the link to the electronic supplementary material.

Supplementary Material 1

## Data Availability

The datasets generated and/or analyzed during the current study are available in the NCBI GenBank repository- https://www.ncbi.nlm.nih.gov/nuccore/OK482790.

## **Abbreviations**

*amoA*: Ammonia monooxygenase
CMC: Carboxymethyl cellulose
*Nar*: Nitrate
*Nir*: Nitrite
*NxrA*: Nitrite oxidase
PGPR: Plant growth promoting rhizobacteria
*S. marcescens*: *Serratia marcescens*
