# Supplementary material for: Characterization and genomics identification of key genes involved in denitrification-DNRA-nitrification pathway of plant growth-promoting rhizobacteria (Serratia marcescens OK482790)
Source: BMC Microbiol. 2023 Aug 5;23:210. doi: 10.1186/s12866-023-02941-7 (PMC10403818; doi:10.1186/s12866-023-02941-7)
Supplement: Supplementary file 1 — Supplementary Material 1 [file 12866_2023_2941_MOESM1_ESM.docx]

Supplementary File “Isolation, characterization, and genomics identification of key genes involved in denitrification-DNRA-nitrification pathway of Serratia marcescens OK482790 as a plant growth-promoting rhizobacteria from lupin rhizosphere”.


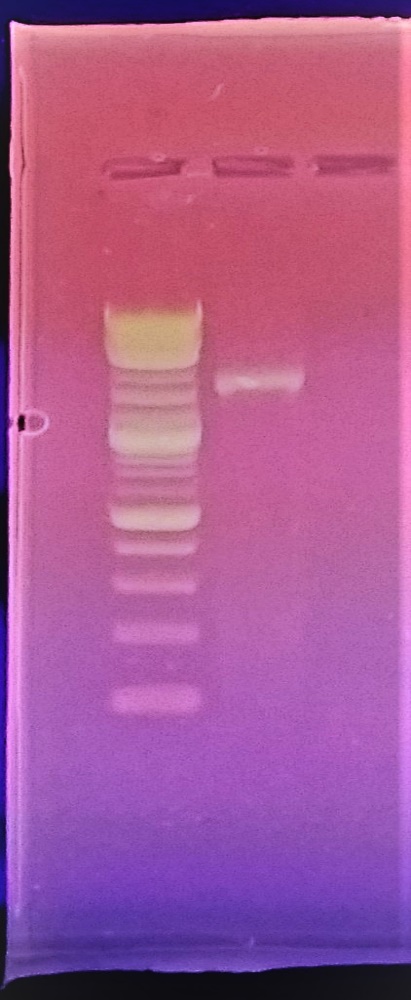


1KB+ Sma -ve

**Supp 1:** Full length Gel electrophoresis image for 1500 bp of 16S rRNA encoding gene PCR fragment. Sma refers to the amplified product, -ve refers to negative control, no amplifications confirm no PCR contamination. 1kb+ refers to DNA ladder (*BioLabs*). (**Supplementary for Figure 1b**).


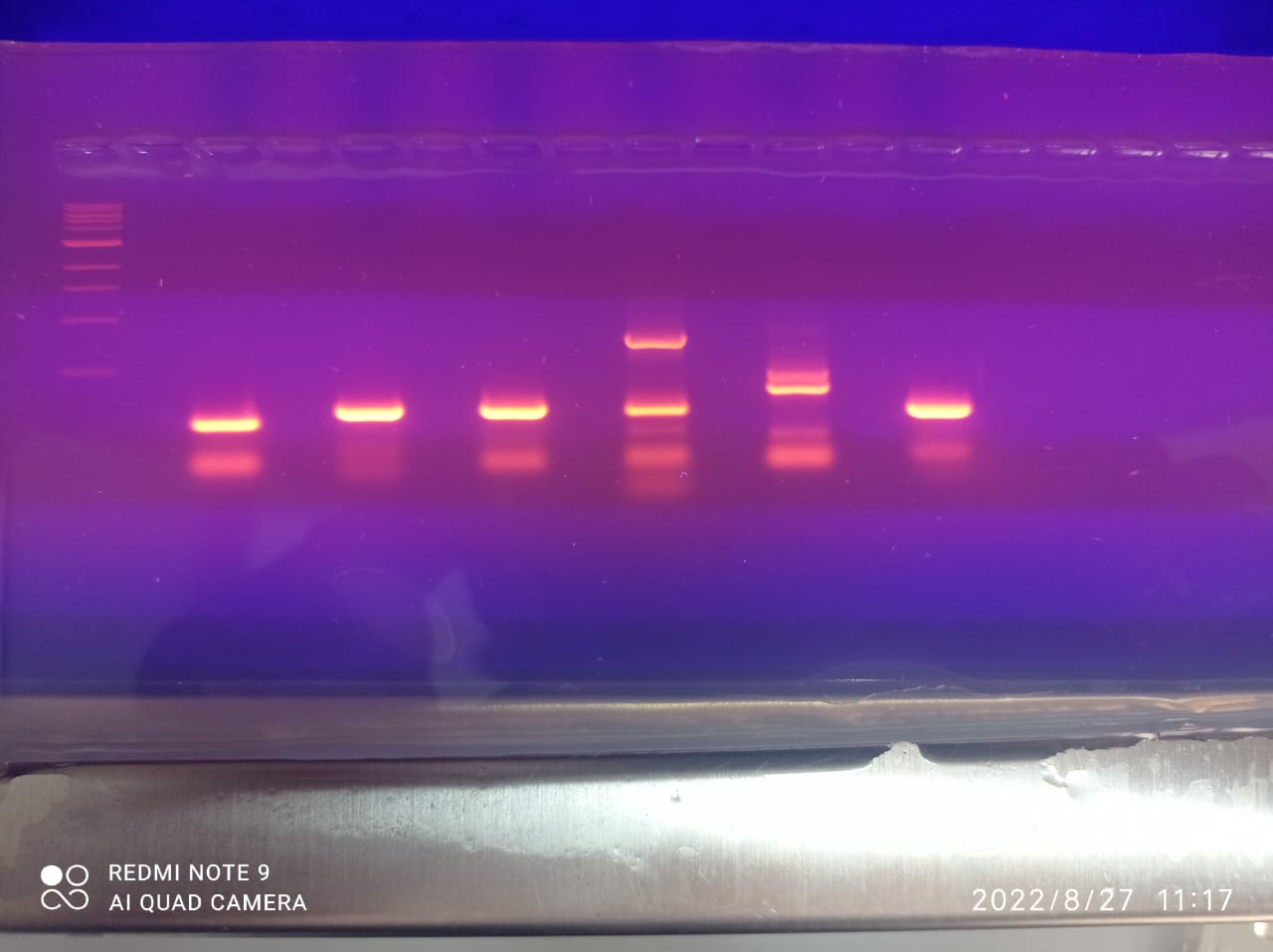


**Supp 2:** Full length image for Agarose gel electrophoresis for PCR products of six nitrogen cycling related genes amplified from *Serratia marcescens* (OK482790); lanes 1 to 6 refers to the amplified products of NarK, NarQ, NarX, NTR, NapC/NirT and oxyred genes respectively, 1KB refers to DNA ladder. The expected product sizes are indicated. **(Supplementary for figure 2**)
